# Mitochondrial DNA as an initiator of tumorigenesis

**DOI:** 10.1038/cddis.2016.77

**Published:** 2016-03-31

**Authors:** W T Y Lee, J C St. John

**Affiliations:** 1 Centre for Genetic Diseases, Hudson Institute of Medical Research, 27-31 Wright Street, Clayton, VIC 3168, Australia; 2 Department of Molecular and Translational Science, Monash University, Clayton, VIC 3168, Australia

For a long time, it was perceived that the mitochondrial genome (mtDNA) was primarily associated with energy production, as it encodes key genes associated with the electron transfer chain, which produces the vast majority of cellular ATP through oxidative phosphorylation. However, the mitochondrial genome is taking an ever-increasingly important role is cellular differentiation, cell fate and, thus, cell function.

Previously, we have shown that copy number of the maternally inherited mitochondrial genome is strictly regulated during development. Indeed, oocytes, embryos and stem cells regulate their mtDNA copy number so that, once cells commit to a specific lineage, they increase their mtDNA copy number in a cell-specific manner.^1, 2, 3^ This enables cells to meet their needs for ATP in order that they can support their specialized functional requirements. For example, neurons have a high requirement for ATP generated through OXPHOS to generate action potentials and mediate neurotransmitter activity. Indeed, in undifferentiated cells, the control of mtDNA copy number is a tightly regulated process as these cells establish the mtDNA set point, which is defined as a small number of mtDNA copies per cell that remains constant until cells initiate differentiation.^1, 2^ Cells then use this population of mtDNA as a template for replication as differentiation ensues. However, cancer cells fail to undergo these transformations as they are unable to mediate mtDNA replication.^4^

Increasing evidence now points to the regulation of mtDNA, either through the number of mtDNA copies^5^ or specific mutations within mtDNA,^6^ as key factors associated with the onset of tumorigenesis. In our paper published in *Cell Death Discovery*, we have used three experimental models to show that depletion of mtDNA directly affects the initiation and progression of tumors *in vivo*.^7^ In each of the three different malignant tumor models, glioblastoma multiforme, multiple myeloma and osteosarcoma, cells with extensively depleted levels of mtDNA had compromised initiation or a significant delay in the onset of tumorigenesis or complete failure to establish tumorigenesis. We further demonstrated that, in those tumors arising from extensively mtDNA-depleted cells, mtDNA was recovered as part of the process leading to the onset of tumorigenesis. This is achieved by the extensively depleted cells undergoing DNA demethylation of the nuclear encoded mtDNA-specific polymerase (*POLGA*),^3^ which is otherwise hypermethylated, and enhances the replication of mtDNA. Furthermore, cells unable to replenish their own mtDNA recruited mtDNA from the surrounding tissues. The acquisition of mtDNA by tumor cells has been previously demonstrated by Tan *et al.,*^8^ whereby they showed that mtDNA was acquired from the surrounding cells of the same species. Our study, however, shows that this can also occur between species, namely human cells acquiring mouse mtDNA but this likely serves primarily to mediate the initiation of tumorigenesis (Figure 1). Indeed, the human mtDNA replication factors are unable to regulate the replication of mtDNA from a more divergent species such as mouse and with only limited efficiency in closely related non-human primates, such as the chimpanzee. These findings suggest that mtDNA could be a potential therapeutic target for the treatment of various cancers but, in order to avoid relapse of the disease, continuous depletion of mtDNA might be necessary until all tumor cells have been targeted.

We further identified specific changes in mtDNA variants in tumors derived from mtDNA-depleted cells. MtDNA variants differ amongst cancer types, but they were also modulated at different stages of tumor progression in two models with decreases at early progression and increases at late progression in our glioblastoma and osteosarcoma models but an overall reduction in our multiple myeloma model at late progression, suggesting that this is cancer type-dependent. We further discovered that the mtDNA-encoded complex I gene *ND6* is a hotspot for mtDNA variants in tumorigenesis. One plausible explanation is that *ND6* is the only protein-coding gene on the light strand of mtDNA and the last gene to be replicated, increasing the possibility of proofreading errors.

Other studies have suggested that higher numbers of mtDNA variants in cancers are likely to contribute to the disease phenotype and the development of cancer.^6, 9^ Moreover, a previous study demonstrated that mtDNA haplotypes can influence mRNA expression of pluripotent factors in murine pluripotent stem cells,^10^ implying an influence from the mitochondrial genome on the nucleus and phenotype. Could this effect be a potential therapeutic approach for cancers, for instance, by replacing mtDNA carrying pathogenic mutations with the wild-type mtDNA? We found that repopulation with wild-type mtDNA from human neural stem cells in the extensively mtDNA-depleted osteosarcoma cells, however, did not prevent tumorigenesis. Instead, it restored the tumorigenicity to levels similar to that of undepleted osteosarcoma cells and those repopulated with mtDNA from human glioblastoma cells (Figure 1). This provides further support for the crucial role that mtDNA has in the establishment of tumorigenesis. Nevertheless, further work is required with wild-type mtDNA from cell types derived from the same lineage to clarify the impact that mtDNA variants have on tumor development.

We also extended our investigations to the transcriptomic landscape, where we repeatedly observed altered gene expression related to hepatic fibrosis, angiogenesis and RhoGDI signaling pathways in tumors with depleted mtDNA. Moreover, we observed that the repopulation of mtDNA partially restored the expression of numerous genes involved in these pathways. Using in-depth transcriptomic analysis of altered expression in tumors at multiple stages of development, we were able to detect and isolate genes that affect post-translational modification and osteoclastogenesis. These findings emphasize the effects that the mitochondrial genome has on the nuclear genome and provides a new approach to detect specific markers for different types of cancers, especially as certain mtDNA haplotypes are more predisposed to cancer.^6, 11, 12^

Overall, this study contributes to our understanding of the role that mtDNA plays in tumorigenesis. Most importantly, these results demonstrate that, although the chromosomal genome regulates the transcription and replication of the mitochondrial genome and contributes largely to disease phenotypes, mtDNA influences the initiation and progression of tumorigenesis. Consequently, mtDNA is a potential therapeutic candidate.

## Figures and Tables

**Figure 1:**
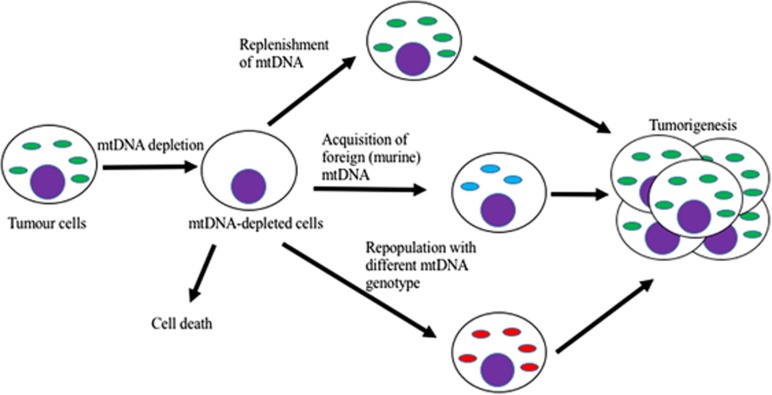
Although depletion of mtDNA copy number shows reduced tumor formation, mtDNA-depleted tumor cells can either replenish their own mtDNA or acquire mtDNA from the surrounding cells, in order to restore impaired tumorigenicity. This is further exemplified through the repopulation of foreign mtDNA in mtDNA-less cells
